# A Case of Cardiac Metastasis from Uterine Cervical Carcinoma

**DOI:** 10.1155/2015/703424

**Published:** 2015-02-24

**Authors:** Kazuhiro Okamoto, Tomoyuki Kusumoto, Noriko Seki, Keiichiro Nakamura, Yuji Hiramatsu

**Affiliations:** Department of Obstetrics and Gynecology, Okayama University Graduate School of Medicine, Dentistry and Pharmaceutical Sciences, 2-5-1 Shikatacho, Kita-ku, Okayama-shi, Okayama 700-8558, Japan

## Abstract

Cases of cardiac metastasis from uterine cervical carcinoma are rare. While they are occasionally found on autopsy, antemortem recognition is extremely rare. We confirmed a case of cardiac metastasis from cervical carcinoma antemortem, because we observed a decrease in platelet count during the course of treatment. The patient was a 27-year-old woman diagnosed with stage Ib1 uterine cervical carcinoma. Radical hysterectomy with pelvic lymphadenectomy was performed. Para-aortic lymph node metastasis was detected on positron emission tomography/computed tomography (PET-CT). Adjuvant chemotherapy was started, and most of the metastatic lesions disappeared. Pelvic lymph node recurrence was suspected on PET-CT during continued chemotherapy; therefore, treatment was shifted to radiation therapy. Tumor shrinkage was recognized, and the initial therapy was completed. A noticeable decrease in platelet count was recognized seven months after treatment. Multidetector CT was performed, and an intracardiac tumor was detected. The patient did not desire any further treatment. She died three weeks after the intracardiac tumor was confirmed. Few previous autopsy studies have reported cardiac metastasis from cervical carcinoma. Thus, it is necessary to consider the possibility of cardiac metastasis for patients diagnosed with terminal cervical carcinoma.

## 1. Introduction

Although cases of cardiac metastasis from uterine cervical carcinoma are occasionally recognized, they are rarely detected before death. Here, we present a case of suspected cardiac metastasis from uterine cervical squamous cell carcinoma. Systemic examinations were performed owing to a decrease in the patient's platelet count. Cardiac metastasis was later diagnosed on autopsy. This report describes this case together with the findings from other literatures.

## 2. Case Presentation

**Figure 1:**
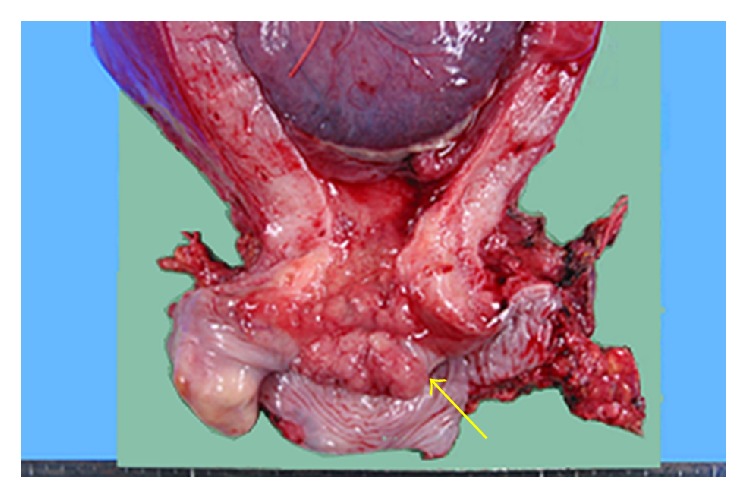
Cervix at operation. The tumor is indicated by the arrow.

The patient was a 27-year-old woman with four previous pregnancies resulting in two births and two abortions. She had no history of appreciable disease. She was diagnosed with class IIIa high-grade squamous intraepithelial lesion by a cervical smear conducted at 14 weeks of pregnancy and diagnosed with squamous cell carcinoma by punch biopsy. She was referred to our hospital at 16 weeks and 6 days of pregnancy for further examination and treatment. No clear macroscopic abnormality was recognized by colposcopy. She was admitted for conization at 18 weeks and 3 days of pregnancy. An exophytic papillary tumor was observed by colposcopy at the time of admission; it may have grown rapidly before admission. We fully explained to the patient and family members that the cancer had advanced and when the baby would be able to survive outside the uterus if the patient continued the pregnancy. The patient and her family desired surgical treatment and termination of the pregnancy. A radical hysterectomy with pelvic lymphadenectomy was conducted at 19 weeks and 0 days of pregnancy (Figure 1).

Postoperative pathological diagnosis revealed the following: uterine cervical carcinoma (pT1b1N1MX), squamous cell carcinoma with a keratinizing type, lymph vascular space positive for invasion, vaginal stump negative for invasion, and lymph nodes metastases (right external iliac lymph nodes, right inguinal lymph nodes, and right obturator lymph nodes).

Metastasis to the para-aortic lymph nodes was detected on positron emission tomography/computed tomography (PET-CT) performed postoperatively. Additional treatment options were discussed and explained. The patient and her family desired treatment that could be administered by visiting the hospital; therefore, chemotherapy was chosen. Weekly TN therapy (taxol 80 mg/m^2^, nedaplatin 25 mg/m^2^, i.v., once weekly) was conducted. Most of the metastases disappeared on PET-CT three months postoperatively and after the completion of nine cycles of chemotherapy. The medical effect of the treatment was judged as PR and chemotherapy was continued. FDG accumulation was noted in the left common iliac lymph nodes on PET-CT six months postoperatively and after the completion of 18 cycles of chemotherapy; this region also showed enlargement on CT. The medical effect of treatment was judged as PD, and chemotherapy was terminated. The treatment method was changed to radiation to the pelvis and para-aortic lymph nodes (whole pelvis: 1.8 Gy/fr, 5 fr/week, and 50.4 Gy; 2 Gy/fr for para-aortic lymph nodes to left common iliac lymph nodes, boosted to 10 Gy, 60.4 Gy in total) and the tumor shrunk. The patient did not desire additional medical treatment such as continued chemotherapy; therefore, we followed up the patient through outpatient visits without treatment.

FDG accumulation was recognized in the para-aortic lymph nodes and both common iliac lymph modes by PET-CT 10 months postoperatively (two months after the completion of radiation treatment). Although the patient had been informed about her condition, she was nearly asymptomatic; she and her family desired to continue follow-up on an outpatient basis.

**Figure 2:**
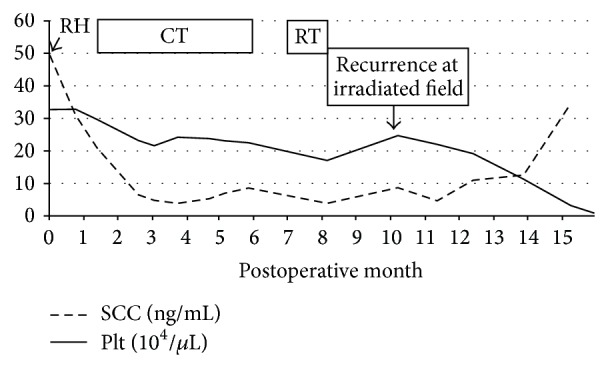
Tumor markers and platelet counts postoperatively. RH: radical hysterectomy, CT: chemotherapy, weekly TN (taxol 80 mg/m^2^, nedaplatin 25 mg/m^2^, i.v., once weekly), RT: radiation therapy whole pelvic 50.4 Gy, para-aortic lymphnode 60.4 Gy, SCC: squamous cell carcinoma-related antigen, and Plt: platelet.

Thereafter, she continued to undergo routine examinations and blood tests through outpatient visits, and her platelet count decreased. She was asymptomatic and desired follow-up visits. A remarkable decrease in platelet count from 32,000/*μ*L was observed in a blood test 15 months postoperatively (seven months after radiation treatment) (Figure 2). Although we attempted to persuade her to be hospitalized for detailed examination and treatment, she wished to be examined and treated through outpatient visits. Her PET-CT scan showed the FDG accumulations in the para-aortic lymph nodes and both common iliac lymph nodes remained nearly unchanged; however, an accumulation was detected in the left gluteus. Accumulations were detected in mediastinum and hilar lymph nodes as well, and metastases were suspected. Bone marrow examination revealed normal hematopoiesis, but the result was probably because of the idiopathic increase in platelet consumption. Multidetector CT showed a tumor extending from the right atrium to the right cardiac chamber (longest diameter, 10 cm).

The cardiovascular internal medicine department concluded that a medical procedure would be difficult. The cardiovascular surgery department was also consulted, and tumor removal by surgery was considered. However, careful judgment as to whether the patient's prognosis could be improved after surgical treatment was necessary. The patient and her family were fully informed about the above information and the possible risks of surgery; they did not desire surgery. Therefore, it was decided she would be followed up continuously.

On the 14th day after confirmation of the tumor, she was admitted to the hospital because of generalized weakness and difficulty with oral intake. Her general condition gradually worsened, and she died on the 21st day after confirmation of the tumor (488th day after the onset of initial treatment). Consent was obtained from the family to perform an autopsy. The findings were as follows:

 patient: a 28-year-old woman;
 clinical diagnosis: uterine cervical carcinoma;
 primary diagnosis:
  1. metastases of uterine cervical carcinoma (squamous cell carcinoma: status after the removal of the uterus, condition after chemoradiation therapy): heart, bilateral lungs, soft structure of the left gluteus, and lymph nodes (para-aortic lymph nodes, para-common iliac artery, and paratrachea);
  2. multiple microscopic tumor emboli and hemorrhagic infarctions of bilateral lungs (left: 382 g, right: 426 g).

**Figure 3:**
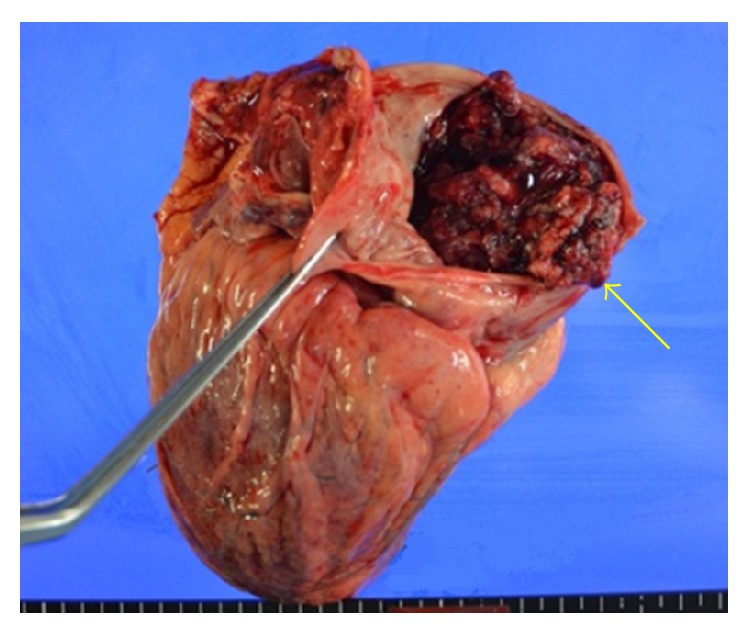
Gross aspect of the heart at autopsy, showing the right ventricle containing the mass (arrow).

**Figure 4 fig4:**
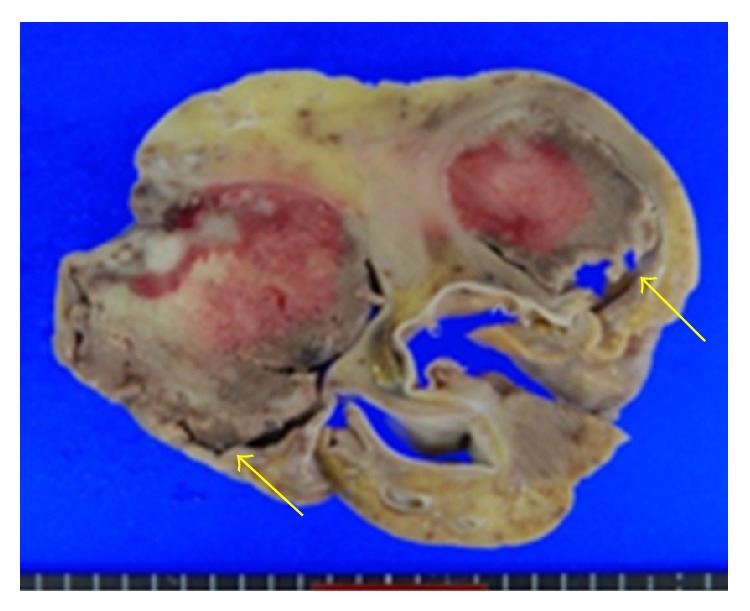
Cross section of the heart, showing tumor involvement of the right ventricle and pulmonary valve.

**Figure 5 fig5:**
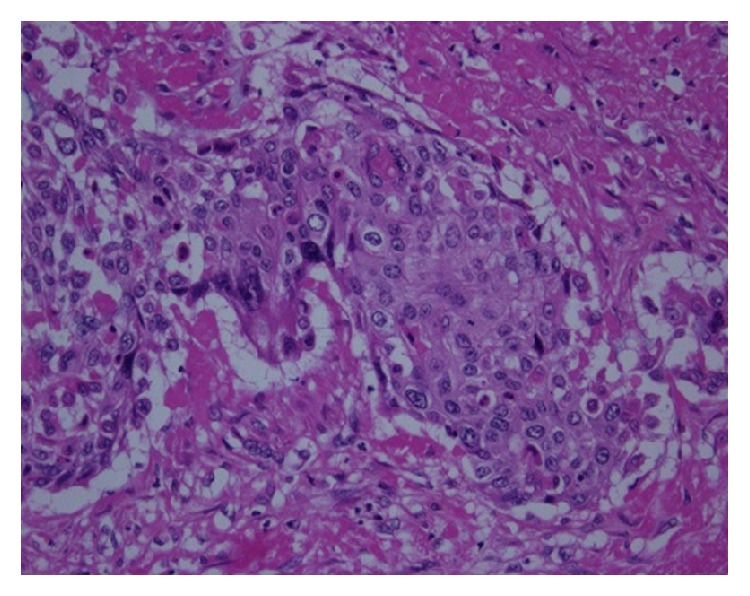
Microscopic view of the tumor, squamous cell carcinoma. Hematoxylin and eosin staining. Magnification ×400.

**Figure 6:**
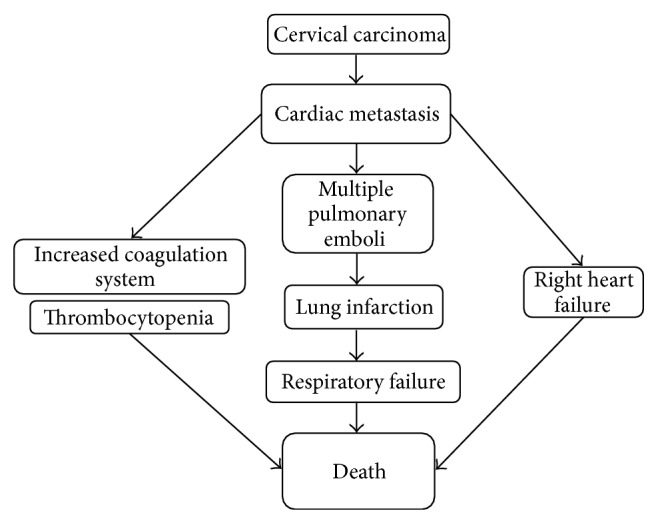
Pathophysiological changes until death.

A metastatic tumor (10 × 6 × 5 cm) extending from the right atrium to the pulmonary artery through the right ventricle was recognized; therefore, intracardiac tumor, tumor embolization, and tumor infarction of the lung periphery were thought to be the cause of death (Figures 3 [Fig fig4] [Fig fig5]–6).

## 3. Discussion

Cases of cardiac metastasis from uterine cervical carcinoma are very rare; less than 40 of such cases have been reported in the literature (Table 1).

Regarding cardiac tumors, metastatic tumors are 40 times more frequent than tumors originating from the cardiac region [1]. According to the autopsy results of cancer patients, the frequency of cardiac metastasis ranges from 1.5% to 21.8% [2, 3]. The primary tumors of cardiac metastases are often malignant melanoma, malignant lymphoma, leukemia, lung cancer, and breast cancer. Cases of gynecological malignancy are relatively infrequent [4, 5] and are rarely diagnosed before death [6]. The prognosis of a metastatic heart tumor is poor; the average life expectancy of patients with this diagnosis is less than six months.

We philologically discussed the cases in which cardiac metastases from uterine cervical carcinoma were found before death. Among the symptoms of 37 cases in which cardiac metastases were found before death, there were 30 (81.5%) cases in which chest symptoms were the most prevalent; among them, the following symptoms were common: sensation of dyspnea, 15/30 (50%); dyspnea, 13/30 (43.3%); chest pain, 10/30 (33.3%); and coughing, 6/30 (20.0%). Echocardiography was used for most diagnoses (Table 1). In all, 29% of the cases were thought to be caused by cardiac metastases, and 16% cases developed cardiac tamponade as a clinical condition [4].

When a patient with uterine cervical carcinoma complains of chest symptoms, it is necessary to confirm the findings by echocardiographic examination and determine whether heart enlargement, an intracardiac space-occupying lesion, or pericardial effusion is present. If pericardial effusion, which could cause chest symptoms, is detected, it is necessary to conduct pericardial drainage and a pathological examination of punctual fluid simultaneously.

In our case, the patient had mild general malaise but only mild symptoms. Therefore, cardiac metastasis from uterine cervical carcinoma was detected through a detailed systemic examination performed because of decreased platelet count. In addition, the following cases were noted in the literature: an abnormality on electrocardiography performed for routine examination for bowel obstruction, a cardiac tumor detected through echocardiography, and a cardiac tumor incidentally detected on gallium scintigraphy performed to confirm the absence of pelvic suppuration as a cause of abdominal pain.

As for the immediate cause of death, cases in which tumor emboli of the lungs led to death have been reported, similar to our case [6, 10, 11]. If the emboli had been found and treated earlier, the symptoms could have been alleviated and the patient's prognosis could have been improved.

Although treatment focused on palliative care in our case, there have been cases in which open-heart surgery was performed and the patients survived for more than two years. Therefore, open-heart surgery is an option to improve survival [14, 27].

In cases of advanced uterine cervical carcinoma, in addition to systemic symptoms including chest symptoms, tumor marker increase, platelet count decrease, and hematogenous metastasis, it is useful to perform other tests such as measurement of D-dimer levels, echocardiography, and a detailed examination for cardiac metastasis using multidetector CT to improve the prognosis and alleviate symptoms.

## Figures and Tables

**Table 1:** Cases of reported antemortem diagnosis of cardiac metastasis from cervical carcinoma.

Year	Authors	Age	Stage	Symptoms	Diagnostic method	Prognosis	Autopsy	References
1967	Dibadj	56	II	SOB	Autopsy	Uncertain	Yes	[7]
1977	Charles et al.	46	IIIb	SOB	Biopsy	8 mo+	No	[8]
1979	Ritcher and Yon	32	IIb	SOB	Echocardiogram	15 d	Yes	[9]
1980	Greenwald et al.	77	IIIb	Dyspnea, SOB, and weakness	Autopsy	5 d	Yes	[10]
1981	Krivokapich et al.	32	IIIb	Chills, dyspnea, fever, and hemoptysis	Echocardiogram and operation	NS	Yes	[11]
1984	Itoh et al.	64	IIb	SOB	Echocardiogram	10 d	Yes	[12]
1986	Hands et al.	43	Ib	Chest pain, lethargy, and nausea	ECG, echocardiogram, and operation	5 mo	No	[13]
1987	Schaefer et al.	28	NS	Edema, SOB, and substernal heaviness	Echocardiogram	2 d	Yes	[14]
1990	Vargas-Barron et al.	55	NS	Aphasia and hemiparesis	Echocardiogram and operation	3 mo+	No	[15]
1990	Malviya et al.	37	IIIb	SOB	NS	3 mo	NS	[4]
1990	Malviya et al.	42	IIIb	Chest pain, cough, dysplasia, and SOB	Biopsy, CT, and echocardiogram	5 d	NS	[4]
1991	Lustig et al.	36	Ib	Chest pain	Echocardiogram	1 mo	No	[16]
1992	Hsu et al.	36	Ib	Cough and dyspnea	Biopsy and echocardiogram	9 mo	NS	[17]
1993	Kountz	28	IIb	Ileus	Biopsy and echocardiogram	3 mo	No	[18]
1993	Nelson and Rose	51	IV	SOB	Biopsy and echocardiogram	4 mo	No	[19]
1993	Nelson and Rose	61	IIIb	Cough and dyspnea	Biopsy and echocardiogram	12 mo	No	[19]
1995	Mohammed S et al.	64	IIIb	Dyspnea, SOB, and weakness	Echocardiogram	3 d	Yes	[20]
1997	Ando et al.	41	IIb	Abdominal pain and dyspnea	Biopsy, gallium scintigram, and MRI	5 mo	Yes	[21]
1997	Batchelor et al.	43	IIb	VF	Biopsy and echocardiogram	1 y+	No	[22]
1997	Batchelor et al.	51	IIb	Chest pain and dyspnea	Autopsy	NS	Yes	[22]
1997	Batchelor et al.	65	NS	NS	Autopsy	NS	Yes	[22]
1998	Lemus et al.	49	IVb	Dyspnea	CT and echocardiogram	7 mo	No	[6]
1998	Lemus et al.	53	Ib	Dyspnea	Echocardiogram and MRI	1 mo	Yes	[6]
1998	Shimotsu et al.	36	Ib	Precordial pain	Biopsy, CT, ECG, echocardiogram, and MRI	NS	NS	[23]
1999	Senzaki et al.	28	Ib	Chest pain and dyspnea	Biopsy and echocardiogram	Less than 1 mo	Yes	[5]
2000	Harvey et al.	44	Ib	None	CT and echocardiogram	8 mo+	No	[24]
2001	Iwaki et al.	49	IVb	Cough, dyspnea, and fever	Biopsy and echocardiogram	2 mo	Yes	[2]
2004	Inamura et al.	58	Ib1	Chest pain, cough, and dyspnea	CT and echocardiogram	4 mo	NS	[25]
2005	Feys et al.	37	IIIb	Cough, fever, SOB, and sweating	Echocardiogram and PET/CT	8 mo+	No	[26]
2005	Saitoh et al.	68	IIIb	Palpitation and SOB	Echocardiogram and operation	5 mo	NS	[3]
2006	Nakao et al.	57	IIIb	Chest pain and dyspnea	Echocardiogram	2 mo	No	[27]
2006	Ferraz et al.	63	NS	Dyspnea and fatigue	Echocardiogram and operation	5 mo+	NS	[1]
2007	Borsaru et al.	42	IVb	Chest pain and respiratory distress	CT, echocardiogram, and operation	NS	NS	[28]
2010	Miller et al.	48	Ib2	Chest pain	Biopsy and MRI	8 mo	NS	[29]
2010	Tomoko et al.	56	Ib2	None	CT, echocardiogram, and PET/CT	25 mo	No	[30]
2013	Byun et al.	32	IIa2	Dyspnea and purpura of extremity	CT, echocardiogram, and operation	13 mo	NS	[31]
2015	Okamoto et al.	27	Ib1	None	Multidetector computed tomography	21 d	Yes	Present case

NS: not stated, SOB: shortness of breath, VF: ventricular fibrillation, ECG: electrocardiogram, CT: computed tomography, PET/CT: positron emission computerized tomography, mo: months, and d: days.

## References

[1] Ferraz J. G. G., Martins A. L. M., de Souza J. F. (2006). Metastatic tumor of squamous cell carcinoma from uterine cervix to heart: ante-mortem diagnosis. *Arquivos Brasileiros de Cardiologia*.

[2] Iwaki T., Kanaya H., Namura M. (2001). Right ventricular metastasis from a primary cervical carcinoma. *Japanese Circulation Journal*.

[3] Saitoh Y., Aota M., Koike H., Nakane T., Iwasa Y., Konishi Y. (2005). Isolated right ventricular metastasis of uterine cervical carcinoma. *Japanese Journal of Thoracic and Cardiovascular Surgery*.

[4] Malviya V. K., Casselberry J. M., Parekh N., Deppe G. (1990). Pericardial metastases in squamous cell cancer of the cervix. A report of two cases. *Journal of Reproductive Medicine for the Obstetrician and Gynecologist*.

[5] Senzaki H., Uemura Y., Yamamoto D. (1999). Right intraventricular metastasis of squamous cell carcinoma of the uterine cervix: an autopsy case and literature review. *Pathology International*.

[6] Lemus J. F., Abdulhay G., Sobolewski C., Risch V. R. (1998). Cardiac metastasis from carcinoma of the cervix: report of two cases. *Gynecologic Oncology*.

[7] Dibadj A. (1967). Intracavitary cardiac tumor secondary to squamous cell carcinoma of cervix. Report of a case and review of literature. *American Journal of Clinical Pathology*.

[8] Charles E. H., Condori J., Sall S. (1977). Metastasis to the pericardium from squamous cell carcionoma of the cervix. *American Journal of Obstetrics & Gynecology*.

[9] Ritcher N., Yon J. L. (1979). Squamous cell carcinoma of the cervix metastatic to the heart. *Gynecologic Oncology*.

[10] Greenwald E. F., Breen J. L., Gregori C. A. (1980). Cardiac metastases associated with gynecologic malignancies. *Gynecologic Oncology*.

[11] Krivokapich J., Child J. S., Warren S. E., Kaufman J. A., Vieweg W. V., Hagan A. D. (1981). M-mode and cross-sectional echocardiographic diagnosis of right ventricular cavity masses. *Journal of Clinical Ultrasound*.

[12] Itoh K., Matsubara T., Yanagisawa K. (1984). Right ventricular metastasis of cervical squamous cell carcinoma. *The American Heart Journal*.

[13] Hands M. E., Lloyd B. L., Hopkins B. E. (1986). Carcinoma of uterine cervix with myocardial metastases associated with chest pain and asystolic arrest. *International Journal of Cardiology*.

[14] Schaefer S., Shohet R. V., Nixon J. V., Peshock R. M. (1987). Right ventricular obstruction from cervical carcinoma: a rare, single metastatic site. *American Heart Journal*.

[15] Vargas-Barron J., Keirns C., Barragan-Garcia R. (1990). Intracardiac extension of malignant uterine tumors. Echocardiographic detection and successful surgical resection. *Journal of Thoracic and Cardiovascular Surgery*.

[16] Lustig V., Vlasveld L. T., Bakker R. H., Schreuder J. E., Mooi W. J., Huinink W. W. T. B. (1991). Intracardiac metastases, report of three cases. *Netherlands Journal of Medicine*.

[17] Hsu J. J., Chang T. C., Hsueh S., Soong Y. K. (1992). Cardiac tamponade resulting from recurrent small-cell carcinoma of the uterine cervix temporarily responding to CE/CAV chemotherapy: report of a case. *Journal of the Formosan Medical Association*.

[18] Kountz D. S. (1993). Isolated cardiac metastasis from cervical carcinoma: presentation as acute anteroseptal myocardial infarction. *Southern Medical Journal*.

[19] Nelson B. E., Rose P. G. (1993). Malignant pericardial effusion from squamous cell cancer of the cervix. *Journal of Surgical Oncology*.

[20] Mohammed S., Khodadoust K. (1995). Carcinoma of the cervix causing massive intracardiac embolus. *Gynecologic Oncology*.

[21] Ando K., Kashihara K., Harada M. (1997). Carcinoma of the uterine cervix with myocardial metastasis. *Gynecologic Oncology*.

[22] Batchelor W. B., Butany J., Liu P., Silver M. D. (1997). Cardiac metastasis from primary cervical squamous cell carcinoma: three case reports and a review of the literature. *Canadian Journal of Cardiology*.

[23] Shimotsu Y., Ishida Y., Fukuchi K. (1998). Fluorine-18-fluorodeoxyglucose PET identification of cardiac metastasis arising from uterine cervical carcinoma. *Journal of Nuclear Medicine*.

[24] Harvey R. L., Mychaskiw G., Sachdev V., Heath B. J. (2000). Isolated cardiac metastasis of cervical carcinoma presenting as disseminated intravascular coagulopathy. A case report. *The Journal of Reproductive Medicine*.

[25] Inamura K., Hayashida A., Kaji Y. (2004). Recurrence of cervical carcinoma manifesting as cardiac metastasis three years after curative resection. *American Journal of the Medical Sciences*.

[26] Feys A., Herregods M. C., Ector H. (2005). Cardiac metastasis from a stage IIIb cervix carcinoma. *Acta Cardiologica*.

[27] Nakao Y., Yokoyama M., Yasunaga M., Hara K., Nakahashi H., Iwasaka T. (2006). Metastatic tumor extending through the inferior vena cava into the right atrium: a case report of carcinoma of the uterine cervix with para-aortic lymph node metastases. *International Journal of Gynecological Cancer*.

[28] Borsaru A. D., Lau K. K., Solin P. (2007). Cardiac metastasis: a cause of recurrent pulmonary emboli. *The British Journal of Radiology*.

[29] Miller E. S., Hoekstra A. V., Hurteau J. A., Rodriguez G. C. (2010). Cardiac metastasis from poorly differentiated carcinoma of the cervix: a case report. *The Journal of Reproductive Medicine*.

[30] Tomoko M., Makoto N., Sachiko O., Takashi U., Hiroyuki K., Yutaka N. (2010). Cardiac metastasis from cervical adenocarcinoma treated with surgical resection and systemic chemotherapy: a case report of a patient who survived for more than 2 years. *The Journal of the Japan Society of Gynecologic Oncology*.

[31] Byun S. W., Park S. T., Ki E. Y., Song H., Hong S. H., Park J. S. (2013). Intracardiac metastasis from known cervical cancer: a case report and literature review. *World Journal of Surgical Oncology*.

